# Development and validation of a nomogram to predict liver metastasis for pancreatic ductal adenocarcinoma after radical resection

**DOI:** 10.3389/fonc.2022.1040411

**Published:** 2022-11-21

**Authors:** Jingshu Tong, Wei Jiang, Shuqi Mao, Shengdong Wu, Caide Lu

**Affiliations:** Department of Hepatopancreatobiliary Surgery, Ningbo Medical Centre Lihuili Hospital, Ningbo University, Ningbo, China

**Keywords:** pancreatic ductal adenocarcinoma, nomogram, liver metastasis, recurrence, radical resection

## Abstract

**Objectives:**

This study aimed to develop and externally validate a nomogram for predicting liver metastasis after radical resection in patients with pancreatic ductal adenocarcinoma (PDAC).

**Methods:**

A total of 247 PDAC patients who underwent radical resection were retrospectively reviewed from January 2015 to March 2022 at Ningbo Medical Centre Lihuili Hospital Eastern Section, and used as a training cohort to develop the nomogram. 83 PDAC patients from the Ningbo Medical Centre Lihuili Hospital Xingning Section were enrolled as the validation cohort. The postoperative liver metastasis was recorded during the follow-up, and the liver metastasis-free survival was defined as the time from operation to the date of liver metastasis diagnosis or death. The nomogram was established based on independent prognostic factors selected by LASSO and multivariate Cox regression model. The performance was assessed using the concordance index (C-index) and calibration curves. The receiver operating characteristic (ROC) curve and decision curve analysis (DCA) were used to determine the clinical utility of the nomogram model.

**Results:**

From the training cohort of 247 patients, a total of 132 patients developed liver metastasis during the follow-up, the 1-, 2- and 3- year liver metastasis-free survival were 52.4%, 43.5% and 40% respectively. The LASSO and multivariate Cox regression analysis indicated that postoperative CA125 (hazard ratio [HR] = 1.007, *p <*0.001), tumor differentiation (HR = 1.640, *p* = 0.010), tumor size (HR = 1.520, *p* = 0.029), lymph node ratio (HR = 1.897, *p* = 0.002) and portal/superior mesenteric/splenic vein invasion degree (PV/SMV/SV) (HR = 2.829, *p <*0.001) were the independent factors of liver metastasis. A nomogram with independent factors was developed and the C-index was 0.760 (95% confidence interval [CI], 0.720-0.799) and 0.739 (95% CI, 0.669-0.810) in the training and validation cohorts, respectively. The areas under curve (AUC) of the nomogram at 1-, 2- and 3-year were 0.815, 0.803 and 0.773 in the training cohort, and 0.765, 0.879 and 0.908 in the validation cohort, respectively, higher than those in TNM stage. Decision curve analysis (DCA) analysis revealed that the nomogram model provided superior net benefit in clinical utility. Liver metastasis-free survival curves showed a significant discriminatory ability for liver metastasis risk based on the nomogram (*p <*0.001).

**Conclusions:**

The nomogram showed high accuracy in predicting liver metastasis for PDAC after radical resection, and may serve as a clinical support tool to guide personalized and prescient intervention.

## Introduction

Pancreatic ductal adenocarcinoma (PDAC) is the 12th most common malignancy and the 7th leading cause of cancer mortality, as one of the most intractable malignant neoplasms worldwide (1). Due to its extremely aggressive nature, radical resection is the only chance for long-term survival for patients with PDAC. However, even after radical resection, most patients still have tumor recurrence or metastasis, resulting in 5-year survival of only 12% to 27%, negatively affecting the curative nature of the operation and the prognosis of PDAC patients (2, 3).

Liver metastasis has the worst prognosis among all recurrence patterns, the median OS is significantly shorter than that of other recurrence patterns (15.4 months vs 17.7-39.6 months) (4). Meanwhile, liver metastasis accounts for the largest proportion of all recurrence patterns, up to 35%-40% of patients (5). Postoperative liver metastasis in patients with PDAC may present a unique biologic characteristic and always indicates a poor prognosis, constituting a key cohort worthy of further study (6). Several stage systems have been used to estimate the overall survival or recurrence-free survival (7, 8), however considering the absence of a prognostic model specifically for liver metastasis after radical resection, it was necessary to develop a predictive model for liver metastasis with an unfavorable prognosis.

In the present study, we developed and externally validated a nomogram to predict the liver metastasis for PDAC after radical resection, which has not been reported in previous studies, aimed to explore the patients with a high risk of liver metastasis after radical resection and potentially assist in clinical.

## Materials and methods

### Patients

The retrospective study consisted of 247 patients who underwent radical pancreatic cancer resection between January 2015 and March 2022 at Ningbo Medical Centre Lihuili Hospital Eastern Section, Ningbo University. The inclusion criteria were as follows: (1) pathology confirmed PDAC, (2) integrated intraoperative and clinical data, (3) enhanced CT/MR performed within 1 month before the operation, and (4) negative final margins with no residual tumor based on pathology. The exclusion criteria were as follows: (1) death within 30 days after the operation, (2) complications with other malignancies, and (3) failure to evaluate the vascular invasion degree from the preoperative imagines or during the operation. To examine the generalizability of the model, the external validation cohort consisted of 83 PDAC patients who underwent radical resection and met the above criteria at Ningbo Medical Centre Lihuili Hospital Xingning Section between January 2016 and August 2021. The study was approved by the ethics committee of Ningbo Medical Center Lihuili Hospital (Approval number: KY2021PJ263). All research procedures complied with the relevant guidelines and regulations. Informed consent was obtained from all patients before inclusion. We confirmed that this study was conducted following the Declaration of Helsinki.

### Assessment of the vascular invasion degree

To assess portal vein/superior mesenteric vein (PV/SMV) and splenic vein (SV) invasion, we recorded the PV/SMV/SV invasion condition in each patient during the operation, evaluated by the chief surgeon. We also review the PV/SMV/SV invasion on preoperative images, evaluated by two radiologists (**Supplementary Figure S1**). The degree of PV/SMV/SV invasion was assessed as follows (9): (1) PV/SMV/SV without tumor abutment or invasion, (2) PV/SMV/SV invasion <180°, (3) PV/SMV/SV invasion >180°.

For most patients, the intraoperative evaluation of vascular invasion was usually consistent with preoperative CT imaging evaluation, if there was a difference, the intraoperative evaluation was prevail.

### Liver metastasis and follow up

Liver metastasis-free survival was defined as the time from operation to the date of liver metastasis diagnosis, death or the last follow-up. The liver metastasis is essentially a particular pattern of tumor recurrence, so the liver metastasis-free survival is a bit like the term recurrence-free survival (RFS), and we concentrated on liver metastasis in this study. The diagnosis of liver metastasis and other recurrence patterns was based on imaging studies, and rarely tissue confirmation. Information regarding liver metastasis was obtained at regular follow-up.

Patients were followed up until September 2022, and all patients were followed up for more than 6 months unless they died. The median follow-up time of patients from the Ningbo Medical Centre Lihuili Hospital Eastern Section and the Xingning Section were 15.0 (range 3-78) months and 19.0 (range 3-77) months, respectively. In general, patients had at least 1 follow-up by imaging study (CT, MRI or PET/CT) and tumor biomarkers every 3 months for the first year after the operation and then every 3-6 months after the first year. Follow-up was performed in the outpatient clinic or *via* phone call.

### Study variables and operation

The following clinicopathological variables were analyzed: demographic data, biochemical tests, tumor markers, pathological features, vascular invasion degree, operative and adjuvant treatment characteristics. The preoperative biochemical and tumor markers test were performed within 7 days before the radical resection, and postoperative tumor markers were measured at the first follow-up. The lymph node ratio was defined as the proportion of positive lymph nodes in the total examined lymph node. The disease stage was evaluated according to the American Joint Committee on Cancer (AJCC) 8th edition and the 7th edition Japanese Pancreas Society (JPS) derived from tumor-node-metastasis (TNM) staging system (10, 11). Adjuvant chemotherapy was routinely recommended and started within 3 months after the operation if conditions permit.

Resectability evaluation and synchronous liver metastasis exclusion were performed by a multidisciplinary team, based on CT and MRI. Surgical methods included pancreaticoduodenectomy and distal pancreatectomy, resected tissues were pathologically examined in frozen and final sections to confirm negative surgical margins. According to preoperative imaging studies and intraoperative exploration, if the tumor invaded, PV/SMV resection and reconstruction were performed in pancreaticoduodenectomy, invaded SV along with the pancreatic body/tail and spleen resection was performed in distal pancreatectomy.

### Statistical analysis

Continuous variables were presented as mean with standard deviation or median with range, categorical variables were presented as frequencies with percentages. Survival curves were calculated using the Kaplan-Meier method and the Log-rank test. Optimal features were selected using the least absolute shrinkage and selection operator (LASSO) regression, and factors with nonzero coefficients were identified and selected. Independent prognostic factors of liver metastasis were identified by univariate and multivariate Cox proportional hazards regression. Subsequently, a nomogram was developed to predict the probability of 1-, 2-, and 3-year liver metastasis-free survival rates after the operation. The performance was evaluated based on the discriminating ability (discrimination) and accuracy of point estimates of the survival function (calibration) with 1000 time bootstraps, and to calculate a relatively corrected concordance index (C-index). The area under curves of the receiver operating characteristic (ROC) curves were calculated and compared with TNM stage, to validate the nomogram model performance. The clinical utility of the nomogram was investigated using the decision curve analysis (DCA), by quantifying the net benefits along with the increase in threshold probabilities. Each patient had a total risk score for risk stratification of liver metastasis according to the nomogram model. Patients were divided into different risk groups (low-; moderate-; high-) with the cut-off points automatically calculated using X-tile software (version 3.6.1; Yale University, New Haven, CT, USA) (12), and further applied to the validation cohort, and the respective Kaplan-Meier curves were constructed.

All statistical analyses were conducted using SPSS software version 24.0 (IBM Corporation, 2020, USA) and R software version 3.6.2 (http://www.r-project.org/). *p* < 0.05 was considered statistically significant.

## Results

### Patients characteristics in the training and validation cohorts

The training cohort consisted of 247 patients who underwent pancreatic cancer resection and had histologically confirmed PDAC at Ningbo Medical Centre Lihuili Hospital Eastern Section, Ningbo University between January 2015 and March 2022. A total of 132 patients developed liver metastasis during the follow-up, and the 1-, 2- and 3- year liver metastasis-free survival were 52.4%, 43.5% and 40% respectively. The validation cohort consisted of 83 eligible patients who underwent radical resection at the Ningbo Medical Centre Lihuili Hospital Xingning Section between January 2016 and August 2021, a total of 46 patients developed liver metastasis, the 1-, 2- and 3- year liver metastasis-free survival were 56.6%, 45.0% and 43.5%, respectively. All clinicopathological characteristics of patients in the training and validation cohorts were summarized (**Table 1**). The patients with liver metastasis may be accompanied by other patterns of recurrence, the specific recurrence patterns of postoperative liver metastasis were summarized (**Table 2**). There was no difference in overall survival between the patients with only-liver metastasis (14.0 months, 95%CI, 11.323-16.677) and the patients with other multiple recurrence (12.0 months, 95%CI, 1.653-22.347, *p*=0.871).

**Table 1:** Clinicopathological and treatment characteristics of PDAC patients in the training and validation cohorts.

Characteristic	Training cohort (n=247)	Validation cohort (n=83)
Age, years, mean ± SD	67.2 ± 9.5	64.5 ± 10.2
Sex, (%)		
Male	137 (55.5)	44 (53.0)
Female	110 (44.5)	39 (47.0)
BMI, kg/m^2^, mean ± SD	22.4 ± 2.8	22.8 ± 2.7
TBIL, umol/L, median (rang)	14.3 (3.6-434.0)	15.6 (1.7-420.0)
DBIL, umol/L, median (rang)	5.2 (0.8-334.8)	7.0 (1.0-380.0)
ALB, U/L, median (rang)	39.3 (27.3-59.0)	39.0 (26.4-54.8)
ALT, U/L, median (rang)	30 (4-723)	30 (10-575)
AST, U/L, median (rang)	26 (9-993)	35 (14-306)
CA199, IU/ml, median (rang)	147.2 (1.2-18722)	21.5 (3.8-4904)
CA125, IU/ml, median (rang)	11.9 (2.0-524.3)	28.4 (1.1-367.0)
CEA, ug/L, median (rang)	2.1 (0.1-66.9)	1.8 (0.1-21.3)
Postoperative CA199, IU/ml, median (rang)	30.1 (1.2-9760)	21.8 (0.7-4708)
Postoperative CA125, IU/ml, median (rang)	24.0 (1.1-198.3)	61.1 (1.4-168.1)
Postoperative CEA, ug/L, median (rang)	1.8 (0.1-55.2)	2.4 (0.1-21.6)
Neoadjuvant chemotherapy, (%)		
Yes	27 (10.9)	8 (9.6)
No	220 (89.1)	75 (90.4)
Tumor location, (%)		
Head/Neck	149 (60.3)	54 (65.1)
Body/Tail	98 (39.7)	29 (34.9)
Surgical path, (%)		
Open	192 (77.7)	66 (79.5)
Laparoscopic	55 (22.3)	17 (20.5)
Tumor size, cm (%)		
>4	91 (36.8)	29 (34.9)
≤4	156 (63.2)	54 (65.1)
Lymphnodes metastasis, (%)		
Yes	120 (48.6)	41 (49.4)
No	127 (51.4)	42 (50.6)
Lymph node ratio, (%)		
≥0.2	54 (21.9)	25 (30.1)
<0.2	193 (78.1)	58 (69.9)
Tumor differentiation, (%)		
Poor	119 (48.2)	49 (59.0)
Well-moderate	128 (51.8)	34 (41.0)
Lymphovascular invasion, (%)		
Present	143 (57.9)	43 (51.8)
Absent	104 (42.1)	40 (48.2)
Perineural invasion, (%)		
Present	213 (86.2)	57 (68.7)
Absent	34 (13.8)	26 (31.3)
Frozen resection margin, (%)		
Positive	33 (13.4)	17 (20.5)
Negative	214 (86.6)	66 (79.5)
Capsule invasion, (%)		
Present	107 (43.3)	39 (47.0)
Absent	140 (56.7)	44 (53.0)
PV/SMV/SV invasion degree, (%)		
Absent	145 (58.7)	47 (56.6)
<180°	53 (21.5)	19 (22.9)
>180°	49 (19.8)	17 (20.5)
Artery reconstruction, (%)		
Yes	4 (1.6)	0 (0)
No	243 (98.4)	83 (100)
Adjuvant chemotherapy, (%)		
Yes	159 (64.4)	67 (80.7)
No	88 (35.6)	16 (19.3)
Morbidity, (%)		
Clavien-Dindo grade 0-II	229 (92.7)	76 (91.6)
Clavien-Dindo grade III-IV	18 (17.3)	7 (8.4)
TNM stage, (%)		
I-IIA	119 (48.2)	37 (44.6)
IIB-IV	128 (51.8)	46 (55.4)

**Table 2:** Recurrence patterns of patients with liver metastasis after the operation.

Liver metastasis patterns (at the date of liver metastasis diagnosis)	Training cohort (n=247)	Validation cohort (n=83)
Liver metastasis only	116 (47.0%)	39 (47.0%)
Multiple recurrences		
Liver+Retroperitoneum	8 (3.2%)	5 (6.0%)
Liver+Locoregional	4 (1.6%)	1 (1.2%)
Liver+Lung	2 (0.8%)	0 (0%)
Liver+Retroperitoneum+Lung	1 (0.4%)	0 (0%)
Liver+Retroperitoneum+Peritoneal+Spleen	1 (0.4%)	0 (0%)
Liver+Bone	0 (0%)	1 (1.2%)
Sum up	132 (53.4%)	46 (55.4%)

### Prognostic factors selection with LASSO analysis in the training cohort

**Figure 1:**
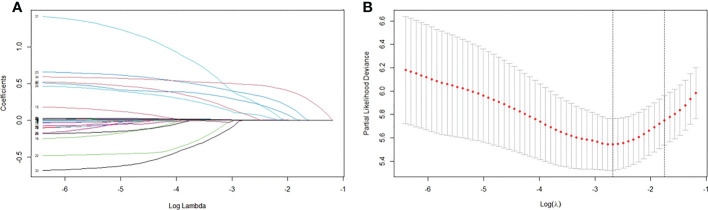
Factors associated with liver metastasis. **(A)** LASSO coefficient profiles of the 34 variables. **(B)** Optimum parameter (Lambda) selection in the LASSO model performed ten-fold cross-validation *via* minimum criteria.

LASSO regression was performed for all 34 clinicopathological characteristics to select the prognostic factors of liver metastasis (**Figures 1A, B**). The neoadjuvant chemotherapy was not an independent prognostic factor of liver metastasis after the operation (HR=1.468, 95%CI, 0.881-2.447, *p*=0.141). The analysis indicated that postoperative CA125, total examined lymph node number, tumor differentiation, lymphovascular invasion, capsule invasion, tumor size, lymph node ratio and PV/SMV/SV invasion degree were associated with liver metastasis after the operation. All significant factors selected from the LASSO regression were further included in the multivariable Cox analysis, and showed that postoperative CA125 (hazard ratio [HR] = 1.007, *p <*0.001), tumor differentiation (HR = 1.640, *p* = 0.010), tumor size (HR = 1.520, *p* = 0.029), lymph node ratio (HR = 1.897, *p* = 0.002) and PV/SMV/SV invasion degree (HR = 2.829, *p <*0.001) were the independent factors for liver metastasis (**Table 3**).

**Table 3:** Univariate and multivariate analysis of predictive factors for liver metastasis in the training cohort.

Variable	Univariate Cox analysis		Multivariate analysis	
	Hazard ratio (95%CI)	*p*-value	Hazard ratio (95%CI)	*p*-value
Postoperative CA125, IU/ml	1.007 (1.003-1.010)	<0.001	1.007 (1.003-1.011)	0.001
Total examined lymph nodes number	1.028 (1.009-1.048)	0.004		
Tumor differentiation				
Poor	Reference		Reference	
Well-moderate	2.168 (1.531-3.070)	<0.001	1.640 (1.126-2.388)	0.010
Lymphovascular invasion				
Present	Reference			
Absent	1.748 (1.219-2.505)	0.002		
Capsule invasion				
Present	Reference			
Absent	1.463 (1.037-2.064)	0.030		
Tumor size, cm				
>4	Reference		Reference	
≤4	2.178 (1.547-3.065)	<0.001	1.520 (1.045-2.210)	0.029
Lymph node ratio				
≥0.2	Reference		Reference	
<0.2	1.844 (1.249-2.722)	0.002	1.897 (1.256-2.866)	0.002
PV/SMV/SV invasion degree				
None	Reference		Reference	
<180°	2.754 (1.806-4.197)	<0.001	2.572 (1.664-3.977)	<0.001
>180°	3.991 (2.641-6.030)	<0.001	2.829 (1.817-4.404)	<0.001

### Construction and validation of nomogram for liver metastasis-free survival prediction

**Figure 2:**
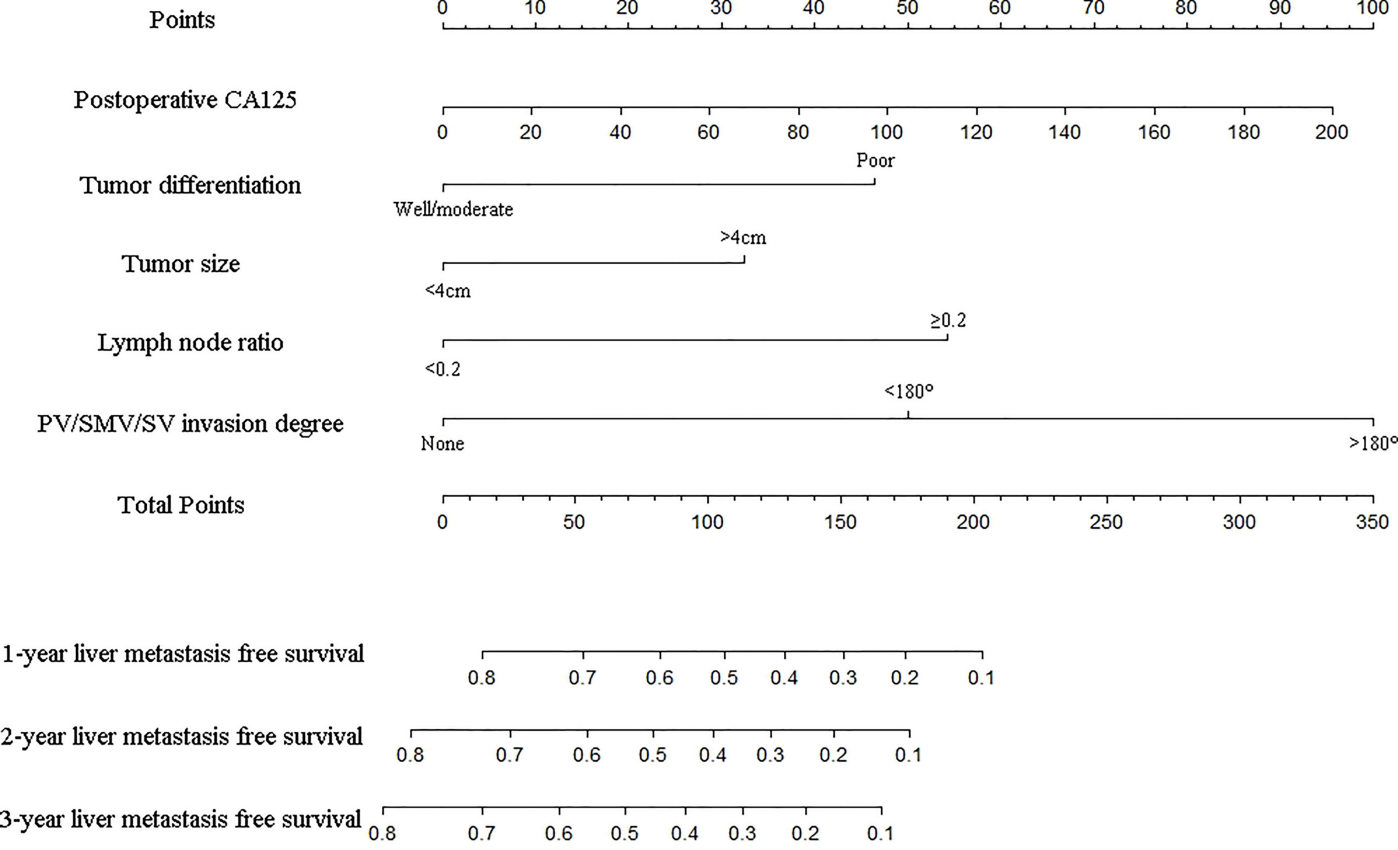
Nomogram for predicting the 1-, 2- and 3-year liver metastasis-free survival in PDAC patients after the operation. The nomogram was established in the training group, with postoperative CA125, tumor differentiation, tumor size, lymph node ratio and PV/SMV/SV invasion degree.

**Figure 3:**
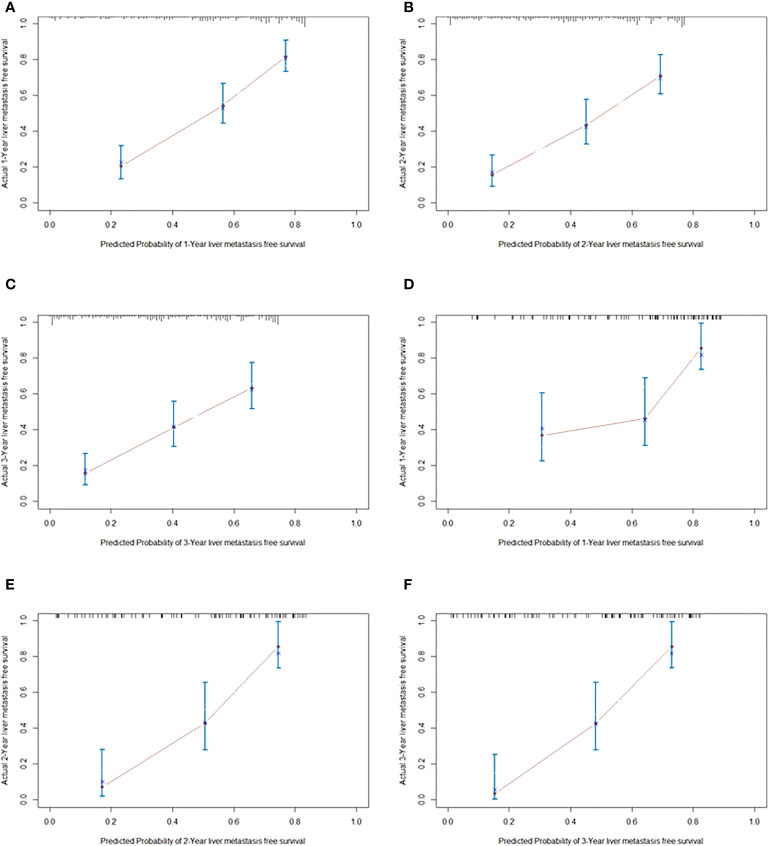
The calibration curves for predicting liver metastasis-free survival at 1 year **(A)**, 2 years **(B)** and 3 years **(C)** in the training cohort, and those at 1 year **(D)**, 2 years **(E)** and 3 years **(F)** in validation cohorts, respectively.

**Figure 4:**
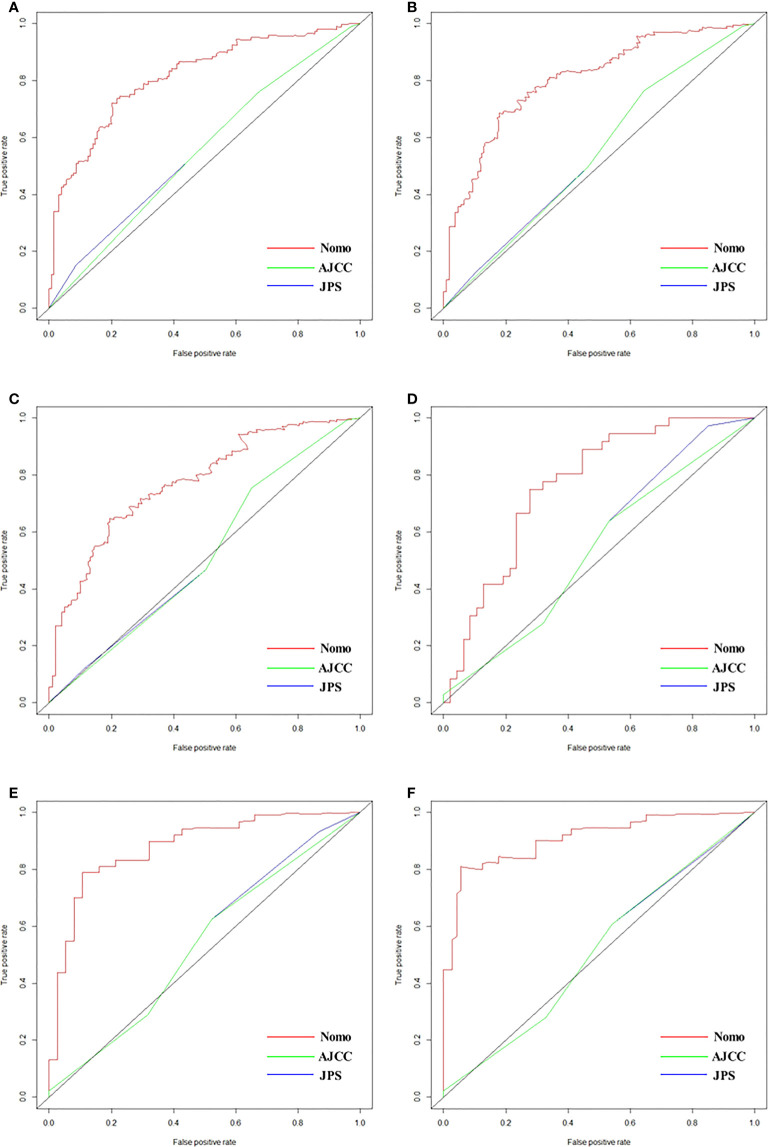
ROCs of nomogram, AJCC and JPS for predicting liver metastasis-free survival at 1 year **(A)**, 2 years **(B)** and 3 years **(C)** in the training cohort, and those at 1 year **(D)**, 2 years **(E)** and 3 years **(F)** in validation cohorts, respectively.

As shown in **Figure 2**, the nomogram was established based on the independent factors of liver metastasis. PV/SMV/SV invasion degree and postoperative CA125 level were the largest contributions to liver metastasis prediction, followed by tumor differentiation and lymph node ratio. The calibration curves showed high agreement between predicted and actual liver metastasis-free survival in both training and validation cohorts (**Figure 3**). The C-indexes of nomogram based on the training and validation cohorts were 0.760 (95% confidence interval [CI], 0.720-0.799) and 0.739 (95% CI, 0.669-0.810), respectively. The AUC of the nomogram at 1-, 2- and 3-year was 0.815, 0.803 and 0.773 in the training cohort, and 0.765, 0.879 and 0.908 in the validation cohort, respectively, all of which were higher than AJCC and JPS of TNM stage system (**Figure 4** and **Table 4**).

**Table 4:** Prognostic performance of different models for predicting liver metastasis after radical resection.

	AUC		
	1 year	2 years	3 years
Training cohort			
Nomogram	0.815	0.803	0.773
AJCC	0.561	0.545	0.522
JPS	0.549	0.549	0.518
Validation cohort			
Nomogram	0.765	0.879	0.908
AJCC	0.530	0.531	0.513
JPS	0.550	0.539	0.511

### Clinical utility of the nomogram

**Figure 5:**
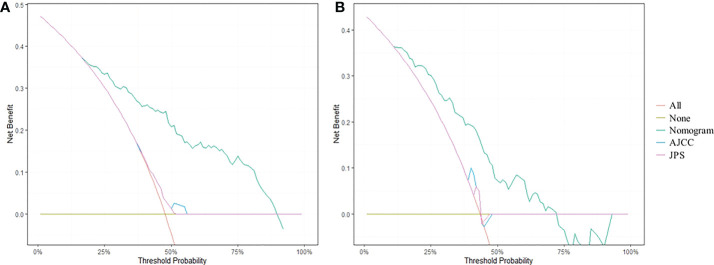
DCA curves for predicting 1-year liver metastasis-free survival based on nomogram as compared with 8th AJCC and 7th JPS stage system in the training cohort **(A)** and the validation cohort **(B)**.

**Figure 6:**
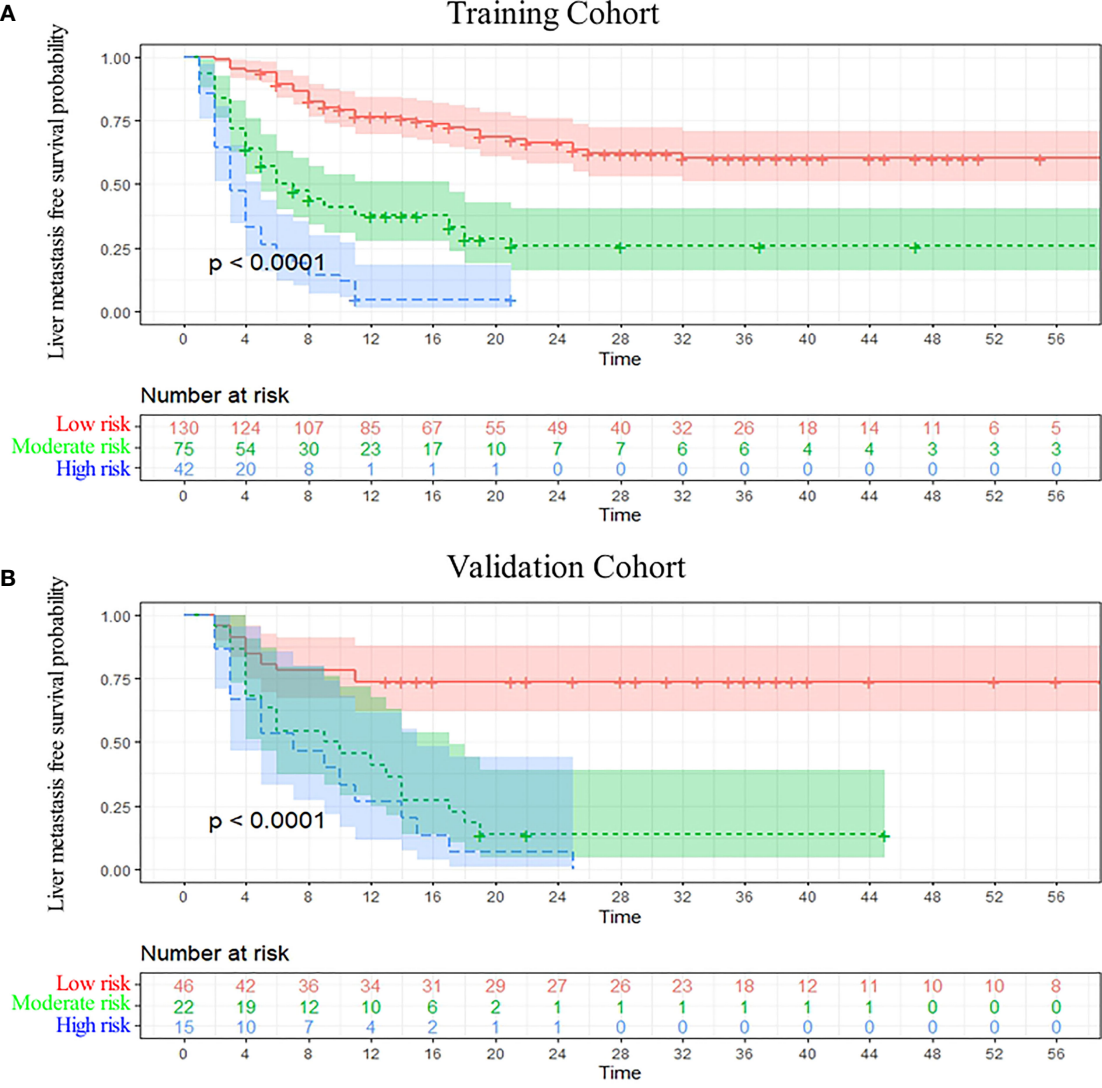
Kaplan-Meier curve analysis. Liver metastasis-free survival curves were stratified by the model risk score in the training cohort **(A)** and the validation cohort **(B)**.

DCA analysis revealed that the nomogram model could provide superior net benefits and exhibited a wider range of threshold probabilities than the AJCC and JPS stage system in both training and validation cohorts (**Figure 5**). Patients were divided into three different risk groups based on the total risk scores calculated by the nomogram models, to validate the predictive abilities of the nomogram for liver metastasis after the operation. The optimal cut-off points were auto-calculated by X-tile software. The risk scores calculated divide patients into the low-risk group (<99.6), moderate-risk group (99.6-160.1) and high-risk group (>160.1). The liver metastasis-free survival rates were calculated in three groups, the results showed a significant discriminatory ability for liver metastasis risk based on the nomogram risk scores (**Figure 6**).

## Discussion

In the present study, we developed and externally validated a nomogram model based on clinicopathological and vascular invasion characteristics, which could be used to predict liver metastasis in patients with PDAC after radical resection. The nomogram model showed superior performance in predicting liver metastasis, with C-indexes of 0.760 (95% CI, 0.720-0.799) and 0.739 (95% CI, 0.669-0.810) in the training and validation cohorts, respectively. As the prognosis of PDAC patients with liver metastasis after radical resection is significantly poor, and currently there is no specifical model for predicting liver metastasis, the present nomogram provided an intuitive and utility tool for guiding the personalized and rational choice of prescient intervention, which is of increased clinical significance.

Liver metastasis is an important feature of PDAC after radical resection, which accounts for the largest proportion and the poorest prognosis among all recurrence patterns, resulting in an increase in mortality (5, 13). Previous study demonstrated that specific patterns of PDAC recurrence result in different survival outcomes, the post progression survival of patients with liver metastasis (4.7months) or multiple-site recurrence (7.2months) had significantly worse when compared to patients with local recurrence (9.7months) or lung metastasis (15.4 months, *p*<0.001) (4). Hishinuma et al. (14) reported that local recurrence is rarely the direct cause of death, instead most patients died of liver metastasis, based on 27 patient autopsies. Previous reports have shown that more than 40% of PDAC patients develop liver metastasis after radical resection (4, 15), similar to the results of this study, but we further focused on liver metastasis throughout the follow-up period, to obtain accurate liver metastasis-free survival in each patient, for developing a more precise and prognostic nomogram model. So, we introduced the term of liver metastasis-free survival, which is a bit like the term recurrence-free survival (RFS), since the liver metastasis is essentially a particular pattern of tumor recurrence, and we only concentrate on liver metastasis during follow-up, for the nomogram development. Morever, the patients with postoperative liver metastasis may also be accompanied by other patterns of recurrence, and we found that there was no significant difference in overall survival between the patients with only-liver metastasis and patients with multiple recurrence, highlighting the malignancy of liver metastasis and the importance of this nomogram.

In the process of developing our nomogram, PV/SMV/SV invasion degree is an important factor, which is not easily measurable as other clinicopathological variables, needed an intuitive and standard classification to define the different invasion degrees. Nakao et al. (16) based on the narrowing of vascular invaded by the tumor, suggested four types of vascular invasion degree: normal, unilateral narrowing, bilateral narrowing and complete obstruction. However this classification has limited capacity in predicting prognosis. Shen et al. (17) reported four types to indicate the relationship between vein and tumor: type 1 (absent), type 2 (mild deformity), type 3 (tethering or stenosis >1/2) and type 4 (obstruction or embolus), this classification can accurately predict the prognosis and similiar to ours. According to the degree of the tumor abutment or invasion, we classified into PV/SMV/SV without invasion, invasion <180°, and invasion >180°, considering both the SMV and SV belong to the portal vein circulatory system, this classification could combine the pancreatic head and body/tail cancer, evaluating the invasion degree in a simple and duplicatable way. As the close adjacent anatomical relationship between the pancreas and PV/SMV/SV, these veins are a common site of direct tumor involvement, but the impact on the prognosis is not clear (18–20). In the present study, PV/SMV/SV invasion was a significant independent risk factor for liver metastasis, 83.7% of patients with vascular invasion >180° developed liver metastasis after radical resection. The “circulating tumor cell (CTC)” hypothesis may explain: that the tumor cells invading the PV/SMV/SV were likely to enter portal vein circulation and metastasize to liver (21, 22). Tien et al. (23) detected the CTCs in portal vein blood obtained during the operation, and found that patients with positive CTCs tended to develop liver metastasis after the operation, supporting the above hypothesis.

Postoperative CA125 level is another independent risk factor of liver metastasis, increased CA125 level after radical resection was an important feature of high PDAC tumor burden and distant metastasis tendency, which indicated the poor curative effect of the operation. Previous study suggested that serum CA125 levels were the most strongly associated with early distant metastasis after pancreatectomy, when compared with other tumor markers such as CA199, CEA, CA242 and CA724. High CA125 levels was consistent with the expression of a “drive” metastasis associated gene signature, which may be the reason for CA125 highly sensitive to liver metastasis (24). Xu et al. (25, 26) also reported that postoperative CA125 level can better predict the prognosis when compared with preoperative tumor markers. Moreover, poor tumor differentiation was associated with liver metastasis as well, in this study, the probabilities of liver metastasis were 35.3%, 50% and 59.5% in the high, moderate and poor tumor differentiation, respectively. A previous large sample study supported our result, indicating that poor differentiation of tumor could promote infiltration and invasion, and contribute to liver metastasis (5). The “intriguing hypothesis” may explain: that poorly differentiated tumors highly expressed epidermal growth factor and E-cadherin, enhanced the ability of liver metastasis (27). Apart from the above risk factors, the nomogram model also covered several risk factors including lymph node ratio and tumor size. Compared with positive lymph node number, the lymph node ratio is a more valuable prognostic indicator, also associated with liver metastasis after radical resection (28, 29). Furthermore, we found that preoperative neoadjuvant chemotherapy was not associated with liver metastasis, which is a regrettable result. We believe that selective bias is the cause: the patients in cohort of neoadjuvant chemotherapy tend to have bigger tumor size and worse vascular invasion degrees, these undesirable tumor characteristic may lead to postoperative liver metastasis, leading to negative result of neoadjuvant chemotherapy.

Compared with the previous traditional nomograms for survival and recurrence prediction, our model can predict liver metastasis after radical resection more specifically and accurately, for early intervention of this unfavorable metastasis. The nomogram achieved a C-index of 0.760 and 0.739 in the training and external validation cohorts, respectively, and the calibration curve indicated the precisely predictive ability of the nomogram in prediction. The present nomogram showed higher AUC and better performance in predicting liver metastasis, when compared with the TNM stage system of 8th AJCC and 7th JPS (10, 11). In addition, DCA analysis indicated that the nomogram could augment net benefits and expose a wider range of threshold probabilities by risk stratification in the prediction of liver metastasis. Furthermore, we calculated the nomogram risk score and compared the liver metastasis-free survival rates, the results showed a significant discriminatory ability for liver metastasis risk based on the nomogram. Liver metastasis possibly represents a unique biological subtype of PDAC (6), personalized follow-up and intervention was needed for the patients with a high nomogram risk score. Randomized clinical trials confirmed that several gemcitabine-based chemotherapies were effective in preventing postoperative liver metastasis and prolonging survival (30). Masayuki et al. (31) reported that hepatic artery infusion chemotherapy can observably increase intrahepatic drug concentration and eliminate tumor metastatic lesions. Additionally, hepatectomy for PDAC patients with postoperative liver metastasis has been proven successful in improving survival (32).

The present study had several limitations. First, liver metastasis was generally based on imaging studies, the tiny hepatic nodules were difficult to identify as metastasis or cyst, limiting the accuracy of the liver metastasis dignosis date. Second, the specific adjuvant chemotherapy regimen after the operation were not included in the variable, making the cohorts relatively heterogenous. In future, a study especially for the patients with/without systemic adjuvant treatment will be established, to explore the effect of systemic adjuvant treatment, as an upgrade to the present nomogram. Third, some differences exist between the training and validation cohorts, but in general, the two cohorts are basically balanced, and the C-index were 0.760 and 0.739, indicating the nomogram has good consistency. Furthermore, a large sample of prospective cohorts is still needed, to further confirm the predictive value.

In conclusion, we developed and externally validated a nomogram to predict liver metastasis after radical resection in patients with PDAC. The nomogram based on clinicopathological characteristics showed great accuracy in predictive performance, and provided an intuitive and utility tool to guide personalized and prescient intervention for patients with a potential risk of liver metastasis.

## Data availability statement

The datasets presented in this study can be found in online repositories. The names of the repository/repositories and accession number(s) can be found below: 1174608081@qq.com.

## Ethics statement

The study was approved by the ethics committee of Ningbo Medical Center Lihuili Hospital (Approval number: KY2021PJ263). All research procedures complied with the relevant guidelines and regulations. Informed consent was obtained from all patients before inclusion. We confirmed that this study was conducted following the Declaration of Helsinki.

## Author contributions

JT and CL proposed and designed the study. SW, W J and SM collected the data. JT and SM analyzed the data, interpreted the results, and drafted the article. All authors contributed to the article and approved the submitted version.

## Funding

Funded by Ningbo medical and health brand discipline (PPXK2018-03).

## Conflict of interest

The authors declare that the research was conducted in the absence of any commercial or financial relationships that could be construed as a potential conflict of interest.

## Publisher’s note

All claims expressed in this article are solely those of the authors and do not necessarily represent those of their affiliated organizations, or those of the publisher, the editors and the reviewers. Any product that may be evaluated in this article, or claim that may be made by its manufacturer, is not guaranteed or endorsed by the publisher.
