# Supplementary material for: Development and validation of a nomogram to predict liver metastasis for pancreatic ductal adenocarcinoma after radical resection
Source: Front Oncol. 2022 Nov 21;12:1040411. doi: 10.3389/fonc.2022.1040411 (PMC9720266; doi:10.3389/fonc.2022.1040411)
Supplement: Supplementary file 1 [file DataSheet_1.pdf]

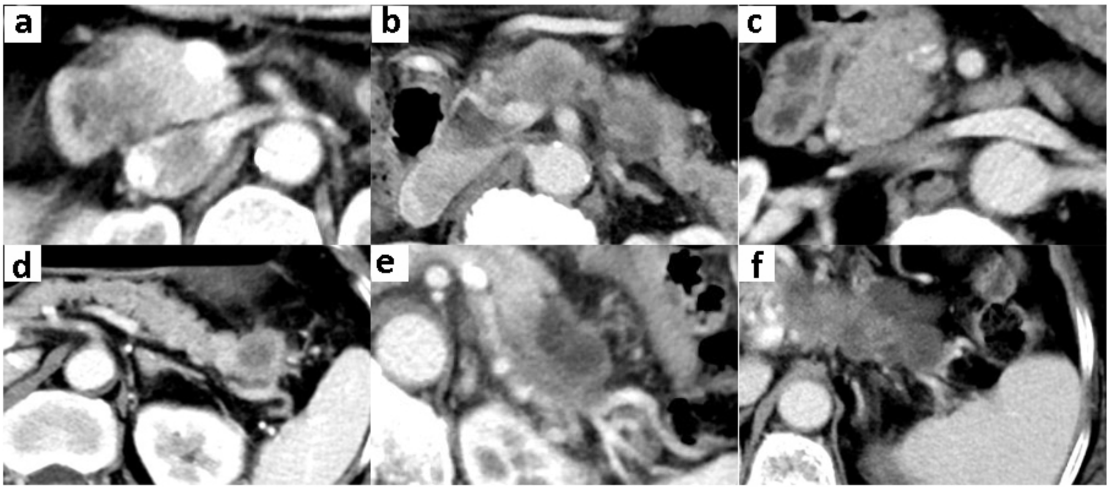

Supplementary Figure S1: Representative findings of PV/SMV/SV invasion in preoperative images. **(a)** No tumor abutment of the PV/SMV, **(b)** Tumor abutment with the PV/SMV and the invasion <180°, **(c)** Tumor invaded the PV/SMV >180°, **(d)** No tumor abutment of the SV, **(e)** Tumor invaded the SV <180°, **(f)** Tumor invaded the SV >180°
